# Intention to Use Contraception: Promises and Pitfalls of Family Planning's Emerging Demand Indicator

**DOI:** 10.1111/sifp.70029

**Published:** 2025-08-11

**Authors:** Jamaica Corker, Ilene S. Speizer, Jean Christophe Fotso, Niranjan Saggurti, Elizabeth Sully

## Abstract

The “intention to use” (ITU) contraception indicator has gained recent prominence as a proposed high‐level success metric for family planning (FP) programs and as a step toward identifying measures that better capture what women want. Although ITU offers advantages over traditional indicators like contraceptive prevalence and unmet need, its elevation as a key programmatic measure requires critical examination. In this commentary, we outline advantages of ITU to measure FP demand and offer critiques and considerations for reliance on ITU as a demand metric for measuring programmatic success. We argue that while ITU may be a step toward more person‐centered measurement, it is not inherently person‐centered. Rather than positioning ITU as an innovative person‐centered breakthrough, we argue it should be considered a transitional measure—a bridge toward more comprehensive indicators that capture the complexities of contraceptive decision‐making. We recognize the current lack of viable alternatives for programs seeking a singular person‐centered measure; when used, ITU should be complemented by additional topline indicators that capture access, agency, and preferences. With declining research funding and data infrastructure disruptions, it is important that ITU complement, not replace, efforts to develop the next generation of FP measurement that meaningfully reflects people's contraceptive realities.

Intention to use (ITU) contraception is a long‐standing family planning (FP) indicator, measured in large‐scale demographic surveys since the 1970s, although its phrasing and timeframe have varied widely across studies (Boydell and Galavotti 2022). Though proposed as a better measure of FP demand for program targeting than unmet need (UMN) as far back as 1997 (Ross and Heaton 1997), ITU historically has not played a central role in FP programming. The recent adoption of ITU as a key demand measure by the Gates Foundation (Gates Foundation 2024), however, has renewed interest in ITU. This positioning of ITU as a primary measure of demand in place of UMN has been presented as joining the “broader movement toward asking women about their desires, preferences, and intentions when it comes to contraceptive use” (Gates Foundation 2024), framing it, even if indirectly, as part of the field‐wide shift toward person‐centered FP measurement. Although ITU may better capture individuals’ own expressed preferences when compared to contraceptive prevalence or UMN, its classification as truly person‐centered remains debatable. As influential donors and organizations begin to use ITU to guide funding and evaluate impact, there is a need for critical reflections on the measure's advantages, limitations, and framing as a person‐centered measure.

## CURRENT MEASUREMENT

Using the term “intention” to describe women's responses to questions about future contraceptive use may overstate a woman's actual “intent,” plan, or desire. The phrasing used in the two most influential global FP surveys, the Demographic and Health Survey (DHS) and Program Monitoring for Action (PMA), ask women “Do you think you will use a contraceptive method to delay or avoid getting pregnant at any time in the future?” The label “intention” stems from variable naming rather than the content of the survey question itself: current nonusers are asked what they think they may do in the future, but do not “state an intent to use” directly or say what they “actually want” (Gates Foundation 2024). For example, women may say they think they are likely to use contraception even if they do not want to or say they do not think they will use a contraceptive method because they do not think they can access any (or the one they want). The term “intention” thus implies a level of decisiveness that may not reflect the nature of the survey question or how women interpret it. The primary value of the core ITU question may in fact lie in identifying the proportion of women who do not think they will use contraception at *any* point in the future (i.e., “no intention”).

In addition to the core ITU question, PMA includes two follow‐up questions: “When do you think you will start using a method?” and “What method do you think you will use?”^1^ PMA's timing question enables construction of a time‐bound ITU variable (e.g., ITU within 12 months). Including a time frame—especially one focused on the near future—offers a closer approximation of implementation intention, which Boydell and Galavotti (2022) found to be more strongly associated with subsequent use than the broader “at any point in the future” phrasing, suggesting it may reflect precontemplation more than true intention. The method‐specific follow‐up allows for tracking of not only whether a woman with ITU goes on to use a method but also whether she adopted the method she thought she would use. This arguably gets closer to measuring method preference, though there is some ambiguity in the question, as the method a woman thinks she will use may be constrained by knowledge or access and may be different from the one she prefers.

## ITU AS A MEASURE OF CONTRACEPTIVE DEMAND

This shift to using ITU as a core demand indicator of FP program success reflects an effort to address persistent conceptual and practical shortcomings with the field's long‐standing dominant global indicators—modern contraceptive prevalence (mCP), UMN, and demand satisfied (DS)—that have long faced criticism (Speizer, Bremner, and Farid 2022; Fabic 2022, Rominski and Stephenson 2019; Bradley and Casterline 2014; Pritchett 1996; Dixon‐Mueller and Germain 1992). Conceptually, these indicators either assume contraceptive use as a universally desirable goal (mCP) or ascribe demand or “need” based on misalignment of a woman's fertility intentions and current contraceptive use (UMN and DS). In contrast, ITU asks women directly about potential future contraceptive use, capturing their own prospective estimate of demand. Others have pointed out that proponents argue this makes ITU more person‐centered, aligning with broader shifts in reproductive health toward respecting individual autonomy and agency (Boydell and Galavotti 2022).

Practically, ITU offers a high‐level contraceptive demand indicator that is easily understood, can be measured with established survey questions, and can track and report progress over a shorter time interval (e.g., 12 months) to inform funding and programmatic decisions. Unlike researcher‐designated UMN, ITU provides a flexible definition of “self‐defined” demand for contraception, serving as a proxy for “contraceptive readiness” (Moreau et al. 2019). This enables programs to identify and target women with an expressed future demand for or willingness to use FP. Self‐reported ITU's greater predictive power for future contraceptive use compared to ascribed UMN has been recognized for nearly three decades (Curtis and Westoff 1996; Roy et al. 2003; Callahan and Becker 2014; Johnson et al. 2019) and reinforced by recent PMA data analysis (Sarnak et al. 2020). Thus, ITU provides a more direct estimate of future demand than mCP or UMN and may be more useful for forecasting product and service needs—especially for specific methods.

ITU is also an attractive high‐level indicator as it is a single, easily interpretable metric to assess the success of specific interventions over shorter time horizons. Although there is growing momentum for more comprehensive and person‐centered measures in FP, including measures of preferences, access, and reproductive or contraceptive autonomy, these measures in their current forms may not be feasible for high‐level tracking and reporting across all programs or donor or country portfolios. Compared to other health areas, FP has struggled to articulate a singular success measure, particularly considering criticisms of mCP and UMN. In contrast, ITU offers a more actionable view of an “addressable burden” for FP programs capturing women not currently using contraception but who indicate future interest. Program success can then be gauged by the “actualization” of ITU, avoiding the measurement pitfalls of mCP or additional users, both of which risk attributing changes to program effects when they may result from macro‐level dynamics (e.g., population growth). Finally, unlike UNM, ITU separates fertility intentions from contraceptive intentions; this distinction allows FP programs focused on increasing contraceptive use among women with an ITU to avoid criticism of coercion or population control objectives that can accompany demand creation activities.

## KEY CHALLENGES IN USING ITU AS A MEASURE OF FP DEMAND

Although the shift from UMN to ITU as a key FP demand measure offers advantages, it also presents several critical challenges to its conceptualization and operationalization that must be acknowledged and addressed, including:

• Lack of standardized definition and measurement: “ITU” lacks a clear, consistent definition, with wide variation across surveys and programs in how it is framed and measured, hindering current cross‐context comparability and limiting ITU's utility as a standardized monitoring or evaluation metric. Recent efforts to define and measure ITU—including during the March 2024 International Expert Group Meeting on Assessing Approaches to Demand‐Side Family Planning Measurement with a Reproductive Justice and Rights Framework (IUSSP 2024) and the December 2024 Workshop on the Measurement of Intention to Use (EVIHDAF 2024)—have generated valuable discussion but not yet led to consensus among the stakeholders engaged in the process.
• Overstated person‐centered framing: Although ITU is arguably more person‐centered than UMN, it is *person‐reported* rather than person‐centered. According to Rothschild et al.’s commentary (2025) in this same special issue, a measure can only be considered person‐centered if it meets two criteria: it must (1) reflect an individual's self‐identified values, needs, or preferences, and (2) capture an individual's assessment of the extent to which these have been fulfilled. Rothschild et al. (2025) do not classify ITU as person‐centered but suggest that ITU may satisfy the first criterion by indicating a “self‐identified preference for future contraceptive use” (p. 5). We argue this preference is inferred rather than explicitly expressed and that the current ITU question, ambiguous in how it is interpreted by respondents, ultimately falls short of meeting both of Rothschild et al.’s person‐centered criteria. As such, we argue that ITU is on the pathway toward more person‐centered measurement but is not itself a person‐centered metric.
• Measuring actualization: Measuring the “actualization” of ITU, particularly to identify key facilitators and barriers to translating ITU, requires longitudinal data that can be resource‐intensive and often infeasible without donor support. These challenges are heightened in the current data landscape, particularly in the wake of the DHS defunding and scaling back of PMA funding. Moreover, given the reliance on longitudinal data to track actualization of ITU, assessments may not be feasible at the regional or national level but instead be limited to smaller projects or localized contexts.
• Lack of a clear directionality: Given that most countries and programs are reliant on cross‐sectional data, ITU's utility as a point‐in‐time, cross‐country measure is limited. Even with standardized definitions and time frames, changes in the percentage of women intending to use in the next 12 months are difficult to interpret, offering little insight for comparisons over time. As programs promote contraceptive options, both demand for contraception and ITU may increase, widening the gap between intenders and users. Like UMN, this makes ITU potentially a moving target. Conversely, a decrease in ITU may narrow the gap between intenders and users and could be interpreted as programmatic success, even if driven by factors such as increasing mis/disinformation or partner or community opposition.
• Misapplication of predictive utility: The strong correlation between ITU and future contraceptive use means programs that focus on transitioning women from intention to actual use are targeting a variable already more predictive of future behavior and thus risk over‐estimating programmatic impacts. This is particularly true in the absence of baseline measures or counterfactuals on how many of those with intention would have transitioned to use without program intervention.

## LOOKING AHEAD: RECOMMENDATIONS

Despite its promise, elevating ITU as a key programmatic or global demand measure may inadvertently reinforce a focus on (modern) contraceptive use as the sole desirable outcome, potentially diverting FP funds and programs away from comprehensive, person‐centered, or rights and justice informed FP approaches. While some identified concerns (e.g., variability in ITU's definition and measurement) can easily be resolved by revisions to the current ITU measure, others (e.g., unclear directionality, and potential to overestimate programmatic impact) may pose challenges to current operationalization of ITU as a demand measure and could result in its misapplication. The measure's core concept—centered on contraceptive use—limits its ability to fully capture contraceptive preferences or identify barriers (such as access, knowledge, or agency) among those without a stated ITU contraception. If implemented with a strict focus on modern methods, ITU arguably targets the “lowest hanging fruit”—women already willing or open to modern contraception—while neglecting those facing greater challenges, representing a contraction rather than an expansion of person‐centered FP measurement. These limitations underscore the need to interpret ITU alongside other topline person‐centered indicators (e.g., agency and access), to avoid further narrowing the scope of programmatic success around mCP at a time when the FP field is shifting toward more comprehensive and person‐centered approaches to measurement and programming.

There are also open questions about ITU's universal applicability as an indicator of contraceptive demand or willingness—questions that should be addressed before it is widely used for cross‐country comparisons, even among a limited set of countries. Further evaluation is needed across diverse settings to assess whether patterns of ITU “actualization”—that is, whether intent translates into use—vary by contexts or geographies, and how these patterns are influenced by factors such as prevailing contraceptive use levels, knowledge, availability, and women's agency or control around contraceptive choices. ITU may be more informative of demand and more predictive of future use in some settings compared to others. For example, Moreau et al. (2019) found that incorporating ITU into UMN made a greater difference in low‐fertility settings, suggesting that ITU may better reflect demand where contraceptive use is higher but be less informative in high‐fertility, low‐use contexts.

In critiquing ITU, we also acknowledge that there is currently a lack of viable alternatives for programs seeking a singular person‐centered measure and for high‐level monitoring. Thus, if ITU in its current form is used as a key demand indicator or baseline for measuring programmatic outcomes, it must be situated alongside other topline or contextual indicators that capture and monitor other aspects of access, agency, or preferences, ensuring programs adopt measures that reflect both present and future contraceptive needs of users and nonusers for a more comprehensive approach to meeting individuals’ FP needs.

The FP field must continue working toward a consensus on a new generation of FP measures, including person‐centered measures of FP demand as part of an FP measurement ecosystem. For ITU, the FP field must continue to leverage existing longitudinal data tracking ITU actualization and generate new data on FP desires, choices and behaviors. For instance, current data collection around ITU should play a transformative role in re‐examining and redefining the often‐criticized DHS “reasons for nonuse” categories for current nonusers, particularly for nonusers who report an intention (or willingness) to use. These efforts should also be leveraged to redefine and improve other key FP measures and could facilitate the identification of which factors move the FP field closer to understanding causal pathways that lead to fulfilled contraceptive needs and preferences. With the end of DHS and declining research funding, the global health field must now reconsider how core indicators are measured—creating a substantial challenge alongside a critical opportunity to redefine contraceptive needs and the data needed to support them.

Immediate steps to improve and refine the ITU indicator—even in the current context of constrained data—could include establishing a standardized definition and measurement approach, more comprehensive testing and validation of the ITU question, and a clearer justification for its use as a programmatic tracking tool rather than exclusively as a person‐centered measurement innovation. Stakeholders relying on this indicator should drive the effort around ITU standardization—a process that should extend beyond LMICs to include surveys and research globally, to establish a universal definition and application of “ITU”. Further psychometric examination of ITU would help determine how well the term “intention” aligns with how women interpret the standard ITU questions and clarify the indicator's limitations. This might include undertaking cognitive testing that seeks to determine what respondents understand when reporting an ITU that may elucidate better or more appropriate person‐centered questions to capture latent demand for contraception. Clearly articulating the use case for ITU for program monitoring, distinct from promoting it for its person‐centered properties, will help other donors and programs assess whether or how to consider ITU as a core demand indicator.

Though ITU as a key program demand measure may represent a step toward person‐centered contraceptive measures, its current use should be clearly presented as a stop‐gap measure while the next generation of comprehensive FP topline metrics are developed. Equally important, elevating ITU must not preclude continued investment in improving and developing new FP measures to more accurately capture contraceptive demand and intentions. The FP field needs continued investment in developing person‐centered metrics that prioritize individuals’ diverse reproductive desires and realities. Despite interruptions to much of the FP measurement advancement work with recent funding cuts, efforts are still underway to inform the post‐2030 measurement agenda, including efforts led by FP2030 and the IUSSP scientific panel on Rethinking Family Planning Measurement with a Rights and Justice Lens, as well as on‐going analysis of ITU survey questions from country‐specific surveys and program evaluations. This work can be leveraged to refine ITU and advance the development of other pragmatic, person‐centered indicators that better capture people's reproductive needs and desires and help the field move past its persistent reliance on mCP and UMN.

Those using ITU have a responsibility to clarify its intended use and assess the implications of its broader adoption across the FP field. Using ITU as a primary demand measure involves programmatic trade‐offs that should be acknowledged as part of broader metric selection—not equated with person‐centered progress—as tensions persist between localized, person‐centered data needs and broader monitoring demands. Shifts in measurement practices are often shaped by influential donors and programs—even when metrics are originally selected for internal purposes. Without a clear articulation of ITU's role—as both a tool for targeted program monitoring and a bridge toward more person‐centered approaches—more widespread use of it as a baseline demand measure for focusing on modern method uptake could inadvertently complicate broader efforts to meaningfully assess and meet people's contraceptive needs. For now, those using ITU for program targeting should continue to drive work to refine the indicator, while also advancing new measures better equipped to capture the complexity of contraceptive decision‐making and individuals’ ability to act on their own needs and preferences.

## Footnotes

1 The DHS previously included questions asking women if they expected to use a method in the next 12 months and which method they thought they would use, but these were phased out in later rounds—the 12‐month intention question in Round 5 (beginning 2003) and the method‐specific question in Round 6 (beginning 2008).

## References

[sifp70029-bib-0001] Gates Foundation . 2024. “Listening to Women: Transforming Family Planning Through Contraceptive Choice.” https://www.gatesfoundation.org/ideas/articles/family‐planning‐intent‐use.

[sifp70029-bib-0002] Bradley, Sarah E. K. , and John B. Casterline . 2014. “Understanding Unmet Need: History, Theory, and Measurement.” Studies in Family Planning 45 (2): 123–150. 10.1111/j.1728-4465.2014.00381.x. 24931072 PMC4369378

[sifp70029-bib-0003] Boydell, Victoria , and Christine Galavotti . 2022. “Getting Intentional About Intention to Use: A Scoping Review of Person‐Centered Measures of Demand.” Studies in Family Planning 53(1): 61–132. 10.1111/sifp.12182. 35119110 PMC9303959

[sifp70029-bib-0004] Callahan, Rebecca , and Stan Becker . 2014. “Unmet Need, Intention to Use Contraceptives and Unwanted Pregnancy in Rural Bangladesh.” International Perspectives on Sexual and Reproductive Health 40(01): 4–10. 10.1363/4000414. 24733056

[sifp70029-bib-0005] Curtis, Sian L. , and Charles F. Westoff . 1996. “Intention to Use Contraceptives and Subsequent Contraceptive Behavior in Morocco.” Studies in Family Planning 27(5): 239–250. 8923652

[sifp70029-bib-0006] EVIHDAF . 2024. “BMGF Workshop on Measurement of Intention to Use Family Planning Method.” https://www.evihdaf.com/2024/12/12/bmgf‐workshop‐on‐measurement‐of‐intention‐to‐use‐family‐planning‐method/.

[sifp70029-bib-0007] Dixon‐Mueller, Ruth and Adrienne Germain . 1992. “Stalking the Elusive ‘Unmet Need’ for Family Planning.” Studies in Family Planning 23(5): 330–335. 1475801

[sifp70029-bib-0008] Fabic, Madeleine Short . 2022 “What Do We Demand? Responding to the Call for Precision and Definitional Agreement in Family Planning's “Demand” and “Need” Jargon.” Global Health, Science and Practice 10(1): e2200030. 10.9745/GHSP-D-22-00030. 35294394 PMC8885353

[sifp70029-bib-0009] IUSSP . 2024 “Assessing Approaches to Demand‐Side Family Planning Measurement with a Reproductive Justice and Rights Framework”. IUSSP Expert Group Meeting Report, Paris. https://iussp.org/sites/default/files/RethinkingFP_Mombasa‐Final%20report.pdf.

[sifp70029-bib-0010] Johnson, Natasha A. , Elena Fuell Wysong , Krystel Tossone , and Lydia Furman . 2019. “Associations Between Prenatal Intention and Postpartum Choice: Infant Feeding and Contraception Decisions Among Inner‐City Women.” Breastfeeding Medicine 14(7): 456–464. 10.1089/bfm.2018.0248. 31166698

[sifp70029-bib-0011] Moreau, Caroline , Mridula Shankar , Stephane Helleringer , and Stanley Becker . 2019. “Measuring Unmet Need for Contraception as a Point Prevalence.” BMJ Global Health 4 (4): e001581. 10.1136/bmjgh-2019-001581. PMC6730575 31543991

[sifp70029-bib-0012] Pritchett, Lance . 1996. “No Need for Unmet Need.” Presentation at the Johns Hopkins School of Hygiene and Public Health, Hopkins Population Center Seminar Series.

[sifp70029-bib-0013] Rominski, Sarah D. , and Rob Stephenson . 2019. “Toward a New Definition of Unmet Need for Contraception.” Studies in Family Planning 50(2): 195–198. http://www.jstor.org/stable/45211127. 30741426 10.1111/sifp.12084

[sifp70029-bib-0014] Rothschild, Claire , Kelsey Holt , Funmilola M. OlaOlorun , Julius Njogu , Abednego Musau , and Christine Dehlendorf . 2025. “Person‐Centered Measurement: Ensuring Prioritization of Individuals' Values, Needs, and Preferences Within the Global Contraceptive Measurement Ecosystem.” Studies in Family Planning. 10.1111/sifp.70023 PMC12501692 40551444

[sifp70029-bib-0015] Ross, John , and Laura Heaton . 1997. “Intended Contraceptive Use among Women without Unmet Need.” International Perspectives on Reproductive Health 23(4): 148–154.

[sifp70029-bib-0016] Roy, T. K. , F. Ram , Parveen Nangia , Uma Saha , and Nizamuddin Khan . 2003. “Can Women's Childbearing and Contraceptive Intentions Predict Contraceptive Demand? Findings from a Longitudinal Study in Central India.” International Family Planning Perspectives 29(1): 25–31. 12709309 10.1363/ifpp.29.025.03

[sifp70029-bib-0017] Sarnak, Dana , Amy Tsui , Fredrick Makumbi , Simon Kibira , and Saifuddin Ahmed . 2020. “The Predictive Utility of Unmet Need on Time to Contraceptive Adoption: A Panel Study of Non‐Contracepting Ugandan Women.” Contraception X 2: 100022. 10.1016/j.conx.2020.100022. 32550537 PMC7286181

[sifp70029-bib-0018] Speizer, Ilene S. , Jason Bremner , and Shiza Farid . 2022. “Language and Measurement of Contraceptive Need and Making These Indicators More Meaningful for Measuring Fertility Intentions of Women and Girls.” Global Health Science and Practice 10(1): e2100450. 10.9745/ghsp-d-21-00450. 35294385 PMC8885354

